# Data on non-communicable diseases: A missed opportunity in Pakistan

**DOI:** 10.7189/jogh.13.03045

**Published:** 2023-09-01

**Authors:** Sarim D Khan, Tazeen H Jafar, Kamran Siddiqi, Taimoor Ahmad, Adnan A Khan, Zainab Samad

**Affiliations:** 1 Department of Medicine, The Aga Khan University, Karachi, Pakistan; 2 CITRIC Health Data Science Center, The Aga Khan University, Karachi, Pakistan; 3 Institute for Global Health and Development, The Aga Khan University, Pakistan, Kenya, United Kingdom; 4 Duke-NUS Medical School, Singapore, Singapore; 5 Department of Health Sciences, University of York, UK; 6 Hull New York Medical School, University of York, UK; 7 Akhter Hameed Khan Foundation (AHKF), Islamabad, Pakistan; 8 Research and Development Solutions, Islamabad, Pakistan

In September 2011, the United Nations (UN) adopted the Political Declaration of the High-Level Meeting of the General Assembly on the Prevention and Control of Non-communicable Diseases (NCDs), providing a road map for Member States and the World Health Organization (WHO) to address the NCD epidemic. This Declaration is guided by the WHO Global Strategy for Prevention and Control of Non-Communicable Diseases and its related action plan (WHA61 · 14, 2008) [1,2], whose key components are surveillance, prevention, and management, meant to guide national action responses against NCDs. In 2015, world leaders accepted the Sustainable Development Goal (SDG) 3.4 aim to reduce premature mortality from NCDs by one-third through prevention and treatment, and to promote mental health and well-being by 2030 [1-3]. However, adherence to these commitments has been limited, especially in low- and middle-income countries (LMICs) [4,5], where three-fourths (77%) of the annual 41 million deaths attributable to NCDs occur, and which also bear an inordinate proportion (85%) of NCD-related premature deaths [3].

**Figure 1:**
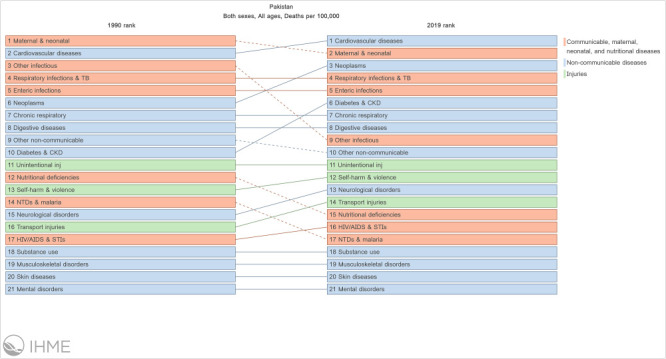
Change in leading causes of death in Pakistan from 1990 to 2019. Downloaded from The Institute for Health Metrics and Evaluation (IHME) at the University of Washington Global Burden of Disease 2019 data visualisation tool.

In Pakistan, an LMIC and the fifth most populous country in the world, NCDs have become one of the leading causes of death. However, the country is still grappling with infectious diseases and high neonatal and maternal mortality: a recent analysis by the Global Burden of Disease 2019 study found neonatal disorders, ischaemic heart disease, and stroke to be among the leading causes of death in Pakistan [6] (**Figure 1**). Lozano et al. [4] estimated the attainment of 2030 targets for the health-related SDGs for 195 countries by calculating a health-related SDG index where a lower index value indicates less progress towards attaining that SDG (**Table 1**).For goal 3.4 (one-third reduction of premature mortality from NCDs), Pakistan lagged behind other South Asian countries with an index value of 23.6 (95% confidence interval (CI) = 13.4-34-0) per 100 000 population for age-standardised death rates due to cardiovascular disease, cancer, diabetes, and chronic respiratory disease in populations aged 30-70 years [4].

**Table 1:** Health-related SDG Index values for specific LMICs for age-standardised death rate due to cardiovascular disease, cancer, diabetes, and chronic respiratory disease in populations aged 30-70 years, per 100 000 population [4]*

Country	Index (95% CI)	Estimated deaths per 100 000
Afghanistan	1.3 (0.0-6.9)	965 · 2 (844.2-1096.8)
Pakistan	23.6 (13.4-34.0)	644 · 1 (539.6-754.3)
India	39.2 (36.7-41.6)	494 · 0 (473.9-508.1)
Bangladesh	44.3 (37.9-50.9)	453 · 7 (407.6-502.7)
Nepal	44.4 (36.5-54.3)	453 · 5 (384.4-513.4)
Bhutan	60.6 (50.0-72.1)	345 · 5 (284.5-411.9)
Sri Lanka	71.4 (61.3-81.5)	287 · 8 (240.9-335.9)

SDG – Sustsainable Development Goal, LMIC – low- and middle-income country, CI – confidence interval

*Indices and individual indicators are reported on a scale of 0 to 100, with 0 representing the worst scores from 1990 to 2030 and 100 reflecting the best during that time.

A key challenge in tackling NCDs is the lack of quality data to understand current disease epidemiology and to inform public and private sector action on NCD nationally. Although surveillance is a major component of the WHO-recommended NCD action plan framework, little work has been done to strengthen national capacity in this area.

The only comprehensive and representative national health survey that collected data on hypertension, diabetes, hyperlipidaemia, and related risk factors was conducted during 1990-1994 by the Pakistan Medical Research Council (PMRC) with technical assistance from the US National Center for Health Statistics [7-9]. Collected almost three decades ago, the data showed an alarming burden of NCDs: 25.0% of adults over the age of 15 years suffered from hypertension, 10.0% had diabetes, and 12.6% had high cholesterol; 34.0% of men and 12.5% of women used tobacco. Furthermore, Jafar et al. [8] reported that 40% of women between the ages of 45-64 years were either overweight or obese. However, no nationally representative survey has been conducted on NCDs in Pakistan during the last three decades.

The extensive, albeit subnational NCD risk factors Pakistan (WHO STEPS) survey conducted in two provinces in Pakistan from November 2013 to April 2014 showed a much higher prevalence of hypertension (53.0%) and highlighted a concern for undetected disease, as an alarming 78.5% of the study population had never had their blood glucose checked [10]. However, three of the five major provinces of Pakistan – Khyber Pakhtunkhwa, Gilgit-Baltistan, and Baluchistan were not included in the survey. This is an important gap, as recent preliminary data made available in April 2022 from the Ehsaas Nationwide Socio-Economic Registry (NSER) revealed that the provinces of Baltistan, Khyber Pakhtunkhwa, and Pakistan-governed Kashmir had the highest prevalence of self-reported chronic diseases, at 66.0%, 50.0% and 49.0% respectively. The nationwide data also showed a prevalence of 18.9%, 14.4%, and 10.5% for cardiovascular disease, diabetes, and hypertension, respectively. The NSER may have only captured partial data on the NCDs, as it collected self-reported data while demonstrating significant socioeconomic inequities across provinces in Pakistan.

Pakistan’s health system response to this alarming data has been inadequate. While Jafar et al. [9] highlighted an appropriate surveillance model with quality data for monitoring as a key response action, no attempt was made to establish it as of yet.

Several surveys are regularly undertaken to track health and its social determinants in the country. For example, Pakistan Social and Living Standards Measurements (PSLM) survey has a health section that is updated yearly, but is focused on children’s immunisation status, reproductive health, and common infectious diseases like diarrhoea. The Pakistan Demographic Health Survey’s (DHS) standard component is conducted every five years. However, it mainly focuses on child and reproductive health indicators and infectious diseases like malaria and HIV, largely ignoring NCDs except for tobacco use as a related risk factor. The DHS survey is funded primarily by the United States Agency for International Development (USAID) along with the Government of Pakistan [11]. The provincial-level Multiple Indicator Cluster Survey (MICS) addresses women and child health (primarily funded by the United Nations Children’s Fund (UNICEF)) and captures data on nutrition-related stunting, but has no NCD indicators [12].

Consequently, many national-level surveys do not track or reflect the heavy burden of NCDs. This may be due to a lack of clear direction from the government on the surveys’ needs-responsivity, and may also reflect the funding bodies’ priorities. Regional endemic infectious diseases are still being prioritised, while the damage NCDs are slowly causing to the Pakistani population is being neglected. Contrastingly, some LMICs have prioritised NCDs in their health surveillance agendas. For example, in Brazil, the Risk Factors for Chronic Diseases by Telephone Interviews (VIGITEL) survey, conducted annually since 2006, utilises mobile-based interviews to assess risk factors for NCDs, such as smoking, alcohol consumption, physical activity, and nutrition [13]. VIGITEL provides invaluable data on NCD risk factors and health indicators in Brazil, helping inform public health interventions and policies. In India, the National Family Health Survey (NFHS), conducted every five years, collects household-level data on the prevalence of NCDs and related risk factors. The survey is nationally representative and receives technical assistance from organisations such as ICF, with funding support from USAID, the United Kingdom Department for International Development, the Bill and Melinda Gates Foundation, the UNICEF, the United Nations Population Fund, and the Ministry of Health and Family Welfare, Government of India [14]. Similarly, the Philippines conducts the Philippine National Nutrition Survey (NNS), an official nationwide survey on nutritional status, diet, and lifestyle-related risk factors for NCDs [15]. Initially conducted in 1978, the NNS has been repeated periodically every five years since 1993. In 2018, it was redesigned as the Expanded National Nutrition Survey (Expanded NNS) and conducted for three consecutive years. These two surveys provide essential data for understanding the nutritional status and lifestyle risk factors associated with NCDs in the Philippines, highlighting the successful implementation of mobile-based, periodic, and nationally representative surveys to assess NCD risk factors and gather important data on health indicators in developing countries. Similar approaches are also employed in other developing countries, enabling the monitoring of NCD risk factors and informing public health interventions. It is imperative that all health data-gathering efforts, especially regular national surveys, are responsive to the rapidly changing health demography of the Pakistani population and intentionally track NCD risk factors and NCD outcomes.

**Figure:**
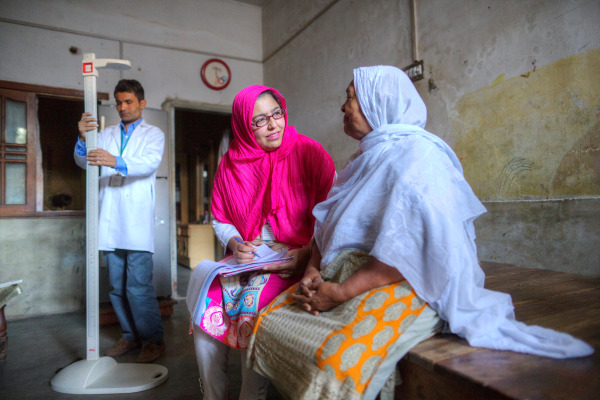
Photo: Taken in 2016 at Issa Nagri, a neighbourhood in Karachi, Pakistan. The research team can be seen filling out a questionnaire, checking blood pressure, and recording body mass indexes (BMIs) of patients in the community. The study was conducted as a part of The Center for Cardiometabolic Risk Reduction in South Asia (CARRS) and the principal investigator (PI) was Dr Masood Kadir from the Department of Community Health Sciences at Aga Khan University (AKU). Source: A photographer named Kohi Marri was hired independently for the project, and as such, the images taken are the property of AKU and can be used freely by AKU employees for dissemination and use in publications.

After the 18^th^ Constitutional Amendment in Pakistan that led to the devolution of health from the federal to the provincial government in 2010, maintaining uniformity of efforts across different provinces has been challenging. Data collection and risk factor reduction efforts have varied across the provinces over the last decade. The 2014-2015 NCD risk factors Pakistan survey did not report disease trends data by province [10]. However, significant differences in the prevalence of hypertension have been identified amongst Pakistani ethnic subgroups after adjusting for confounders [8]. There are certainly socio-ethno-geographic and gender equity elements that need to be tracked across provinces as highlighted by the NSER and other surveys. Thus, there is a need for data at the provincial level to identify regions with higher risk factor and disease burden, allowing the design and implementation of personalised, community-based public health approaches with the respective provincial governments [16].

Collecting quality baseline data on NCD prevalence in Pakistan and doing it consistently is of paramount importance. Funding agencies must realise the need to support research on NCDs and their risk factors to address the country’s dynamic and changing health needs through cost-effective approaches. First, NCD data collection can be joined onto pre-existing PSLM, DHS, and MICS surveys by utilising their existing survey infrastructures (both human and financial) to capture NCD-related health indicators and provide a life-course view of NCDs in Pakistan. Second, existing district health information systems that track maternal and child health indicators of individuals seeking care at the district level health units can also be designed to more comprehensively include NCD risk factors and outcomes. Finally, digital mobile instead of traditional physical paper-based surveys are a potentially unexplored avenue to collect population-level data on NCDs in Pakistan and will greatly reduce administrative and human labour burden. Efforts to enhance the visibility of NCDs are fruitful in an environment where NCD- trained researchers highlight the health and economic impact of NCDs along with their solutions. Their continuous engagement with government and non-government stakeholders is often instrumental in translating research findings to policy changes and implementation of local evidence-based population-level interventions. The media also plays a vital role in stimulating these efforts through awareness raising and engaging with opinion leaders. Apart from data collection, there should also be a focus on strengthening research capacity in NCD and engagement between policymakers, media, and researchers. NCDs and their risk factors can also be incorporated within ongoing national surveys. For example, in a recent engagement between NCD academics and the National Bureau of Statistics, Pakistan, willingness was expressed to incorporate NCD risk factors in the PSLM survey. Finally, the universal health coverage model can stimulate efforts for NCD action through the Non-communicable Diseases and Mental Health National Action Framework 2021-2030 endorsed by the Pakistan provincial and federal governments, along with other relevant stakeholders.

Without data, we do not know how far we are from achieving SDG 3.4 or how to get there. We strongly urge the government of Pakistan and funding agencies involved in health assessment at a national level to add NCDs to their health tracking agenda. The coming generation will pay the price if we fail to rise to this global, time-sensitive challenge.
